# Troponin release following endurance exercise: is inflammation the cause? a cardiovascular magnetic resonance study

**DOI:** 10.1186/1532-429X-12-38

**Published:** 2010-07-02

**Authors:** Rory O'Hanlon, Mat Wilson, Riccardo Wage, Gillian Smith, Francisco D Alpendurada, Joyce Wong, Annette Dahl, Dave Oxborough, Richard Godfrey, Sanjay Sharma, Michael Roughton, Keith George, Dudley J Pennell, Greg Whyte, Sanjay K Prasad

**Affiliations:** 1 Department of Cardiovascular Magnetic Resonance, Royal Brompton and Harefield NHS Foundation Trust, London, UK; 2 ASPETAR Qatar Orthopaedic and Sports Medicine Hospital, Qatar; 3 University of Leeds, Leeds, UK; 4 Department of Sport and Exercise Science, Brunel University, Uxbridge, London, UK; 5 Department of Heart Muscle Disorders, Kings College London, London, UK; 6 R-Squared Statistics, London, UK; 7 Research Institute for Sport and Exercise Science, Liverpool John Moore's University, Liverpool, UK; 8 Centre for Sports Cardiology, Harley Street, London, UK

## Abstract

**Background:**

The aetiology and clinical significance of troponin release following endurance exercise is unclear but may be due to transient myocardial inflammation. Cardiovascular magnetic resonance (CMR) affords us the opportunity to evaluate the presence of myocardial inflammation and focal fibrosis and is the ideal imaging modality to study this hypothesis. We sought to correlate the relationship between acute bouts of ultra endurance exercise leading to cardiac biomarkers elevation and the presence of myocardial inflammation and fibrosis using CMR.

**Methods:**

17 recreation athletes (33.5 +/- 6.5 years) were studied before and after a marathon run with troponin, NTproBNP, and CMR. Specific imaging parameters to look for inflammation included T2 weighted images, and T1 weighted spin-echo images before and after an intravenous gadolinium-DTPA to detect myocardial hyperemia secondary to inflammation. Late gadolinium imaging was performed (LGE) to detect any focal regions of replacement fibrosis.

**Results:**

Eleven of the 17 participant had elevations of TnI above levels of cut off for myocardial infarction 6 hrs after the marathon (0.075 +/- 0.02, p = 0.007). Left ventricular volumes were reduced post marathon and a small increase in ejection fraction was noted (64+/- 1% pre, 67+/- 1.2% post, P = 0.014). Right ventricular volumes, stroke volume, and ejection fraction were unchanged post marathon. No athlete fulfilled criteria for myocardial inflammation based on current criteria. No regions of focal fibrosis were seen in any of the participants.

**Conclusion:**

Exercise induced cardiac biomarker release is not associated with any functional changes by CMR or any detectable myocardial inflammation or fibrosis.

## Introduction

Acute bouts of ultra-endurance exercise may depress global left ventricular (LV) diastolic and systolic function and in the majority of cases this is associated with elevations in circulating serum markers of cardiac damage, including troponin T and I, myoglobin, creatine kinase (CK), CK-MB, and brain natriuretic peptide (BNP) [1-5]. Debate continues as to the clinical significance of changes in cardiac biomarkers and function after acute and chronic endurance exercise. Whether exercise induced cardiac biomarker release is a physiological or pathological process remains a contentious issue. It is likely that acute elevations of cardiac troponins following endurance exercise are physiological owing to the low peak levels recorded and the rapid return to baseline; troponin release may simply be related to cardiac adaptation to exercise where transient myocardial injury acts as a physiological signal for adaptation leading to enhanced structure and function [6]. Animal studies have however demonstrated that endurance exercise can trigger the development of myocardial inflammation and fibrosis and a limited number of post mortem studies have demonstrated both replacement and interstitial fibrosis in the hearts of athletes who have died suddenly [7-9]. A possible mechanism underlying troponin release with exercise and transient cardiac dysfunction may be related to exercise induced myocardial inflammation.

Cardiovascular magnetic resonance (CMR) is the reference standard imaging modality for the assessment of ventricular volumes, function, and mass. Measurements are accurate with no geometrical assumptions about the ventricle and very reproducible [10,11]. Using specialised imaging protocols, CMR can also image focal and global myocardial inflammation and oedema, owing to the relaxation properties of water, using T2-weighted techniques [12-14]. Imaging after intravenous gadolinium can detect regional hyperemia secondary to inflammation and focal areas of myocardial fibrosis [15-17]. The utility of CMR to detect myocardial inflammation has been validated in numerous studies and the subject of a recent white paper from the ACC [18].

We sought to assess the potential relationship between acute bouts of ultra endurance exercise leading to troponin release and the presence of myocardial inflammation and fibrosis using CMR.

## Methods

We studied 17 recreational athletes before and after a marathon run (26.2 miles). Exclusion criteria included the presence of pre-existing cardiovascular or pulmonary diseases, hypertension, diabetes, and peripheral vascular diseases. No participant had taken part in any significant endurance event in the preceding 12 weeks. Cardiac biomarkers (cTnI, NTproBNP) were collected at an initial assessment 24 hrs prior to, immediately after completion of the marathon, and again 6 hours later. Post marathon CMR was performed 6 hours after marathon completion. This time point was chosen based on an assumption that 6 hours would allow a sufficient amount of time for inflammation to develop and be detectable, corresponding with the time when TnI is typically detectable in ischaemic models representing the liberation of these proteins and enzymes from damaged myocytes [19]. Each participant completed a detailed questionnaire detailing their training history (hours training per week, type of training). The study was approved by the Brompton, Harefield and NHLI research ethics committee and all participants signed informed consent.

### Biomarkers

Analysis of cTnI was determined using the TnI-Ultra assay for the ADVIA Centaur (Siemens Healthcare Diagnostics, Frimley, UK). The detection limit of the instrument was 0.006 μg/L, upper limit of 50 μg/L. The claimed 10% CV was 0.03 μg/L with a 99th centile of 0.04 μg/L. The total assay imprecision was 2.7 to 5.3% in the range 0.8 to 27.2 μg/L. NTproBNP was analysed using the Elecsys 2010 system (Roche Diagnostics, Burgess Hill, UK). The assay is an electrochemiluminescent sandwich immunoassay that uses two polyclonal antibodies directed at residues 1-21 and 39-50 of the NTproBNP molecule. The %CV of the assay is 3.2-2.4% from 20.7-585.5 pmol/L (175-4962 ng/L) with an analytical range of 0.6-4138.6 pmol/L (5-35,000 ng/L).

### Cardiovascular Magnetic Resonance (CMR)

CMR data was collected 24 hrs pre- and 6 hrs post-marathon. A standard volumes and LGE sequence was performed on a dedicated scanner (Siemens Avanto 1.5-T, Erlangen, Germany), with full myocardial coverage using an 8-channel phased array coil [20]. Ventricular volumes, function and mass were quantified using customised analysis software (CMRTools, Cardiovascular Imaging Solutions, London, UK) by a blinded single experienced investigator. Papillary muscles were included in the mass and excluded from the volume. Wall motion was analyzed based on the 16 segment AHA/ACC model [21].

Two CMR validated methods to image myocardial inflammation were performed.

#### 1. Myocardial Inflammation

Focal regions of myocardial oedema/inflammation typically appear bright on T2-weighted imaging. Imaging was performed using a breath hold, black blood, T2 weighted triple inversion recovery TSE sequence (STIR) (TR 2 × RR, TE 67 ms, TI 140 ms) in identical SA slices from base to apex. Typical slice thickness was 8 mm, 2 mm gap, and the FOV was adapted per patient. Regional oedema was identified visually. Global oedema is not recognisable to the naked eye, and quantitative analysis was performed by normalising the signal intensity of the myocardium to that of skeletal muscle (typically the latissimus dorsi). Endocardial and epicardial contours were manually drawn and a region within a skeletal muscle was defined in the same slice. The T2 ratio is calculated as follows:

A value of > 1.9 was considered diagnostic of global myocardial oedema/inflammation [14].

#### 2. Regional Hyperaemia

Regional vasodilatation occurs in areas of tissue inflammation and the increased blood volumes in these areas leads to an increased uptake of gadolinium contrast agents during the early vascular phase. To assess this, we performed T1 weighted spin-echo images before and after an intravenous gadolinium-DTPA infusion (Bayer, Germany; 0.1 mmol/kg) (injection rate 2 ml/sec, followed by 15 ml normal saline) in a single mid ventricular short axis slice with a wide field of view to include a substantial portion of skeletal muscle (typically the latissimus dorsi). Imaging was performed within 1 minute of injection and repeated every 30 seconds for 3 minutes. We measured the SI of the myocardium before and after contrast and also SI of neighbouring skeletal muscle. The Early Enhancement or relative gadolinium enhancement (rGE) ratio is calculated by the following formula:

This was deemed to be positive if the ratio was > 4.0

The enhancement of myocardium and skeletal muscle regions of interest being used for the formula above are calculated by:

The ratio of SI increase before and after contrast for myocardium was deemed to be positive for hyperaemia if % SI myocardial increase was > 45% between pre and post contrast images using commercially available software (CMR Tools, London, UK) [22].

### Myocardial Fibrosis

Imaging for late gadolinium enhancement (LGE) to identify fibrosis was performed 5-10 min after contrast injection in identical short-axis planes to cine images using a breath hold inversion-recovery (FLASH) gradient echo sequence [23]. Inversion times were optimized to null normal myocardium. In all patients, LGE imaging was repeated for each short-axis image in two separate phase-encoding directions to exclude artefact. LGE images were analyzed quantitatively by 2 independent readers using customized software (MRI-MASS, Medis, Leiden, Netherlands). In brief, the endocardial and epicardial borders were traced for each short-axis slice. A region of interest (ROI) averaging 50 mm^2 ^was defined within the normal, remote myocardium in an area with uniform myocardial suppression free of artefacts. A multi-pass region-growing algorithm was used to identify the fibrotic boundaries based on the "full width half maximum" technique and myocardial LGE was expressed as present/absent (LGE+,LGE-), as gram mass, and a percentage of total LV mass [24-26].

### Statistics

All data values were presented as mean ± SD. Changes from pre-marathon to post-marathon were analysed using paired t-tests. Relationships between data indices CMR measures of systolic function against serum biomarkers of cardiac damage were examined via Pearson's product-moment correlation analysis. The critical α level was set at 0.05 and all analyses were carried out on SPSS 16.0 software.

## Results

The participants included 17 men (mean ± SD [range]: age 33.5 ± 6.5 years [26-46 yrs], body mass 80 ± 9.2 kg [63-100 kg], height 1.81 ± 0.06 m [1.7-1.93 m]). The mean number of hours trained per week was 7 hours. All 17 runners completed the study protocol (209 ± 19 min; range, 171-240 min), Table 1. Post-marathon testing commenced within 15 min of marathon completion in all participants. Body mass was significantly reduced post-marathon (80 ± 9.2 vs 78.8 ± 8.6 kg, p < 0.001). Heart rate was significantly increased post-marathon (57 ± 8 vs 80 ± 12 beats/min; p < 0.001), and remained significantly elevated 6 hrs post-marathon (57 ± 8 vs 68 ± 11 beats/min; p < 0.001).

**Table 1:** Individual marathon times, peak troponin level, and volumetric indices pre and post marathon.

Subject	Peak TnI	LVEF Pre (%)	LVEF Post (%)	RVEF Pre (%)	RVEF Post (%)	Marathon Time (mins)
**1**	0.11	56	56	53	48	220
**2**	0.13	61	64	62	58	220
**3**	0.06	62	68	63	65	201
**4**	0.03	64	72	67	66	201
**5**	0.03	65	66	57	59	233
**6**	0.05	59	64	63	65	220
**7**	0.03	64	68	71	72	188
**8**	0.03	64	64	59	57	202
**9**	0.2	74	77	62	62	188
**10**	0.02	68	74	65	70	208
**11**	0.05	64	73	60	65	208
**12**	0.01	72	62	73	63	218
**13**	0.43	62	67	55	60	210
**14**	0.03	66	65	65	66	171
**15**	0.03	65	68	56	61	240
**16**	0.03	66	67	61	60	190
**17**	0.06	63	71	63	70	240

### Cardiac Biomarkers

**Figure 1:**
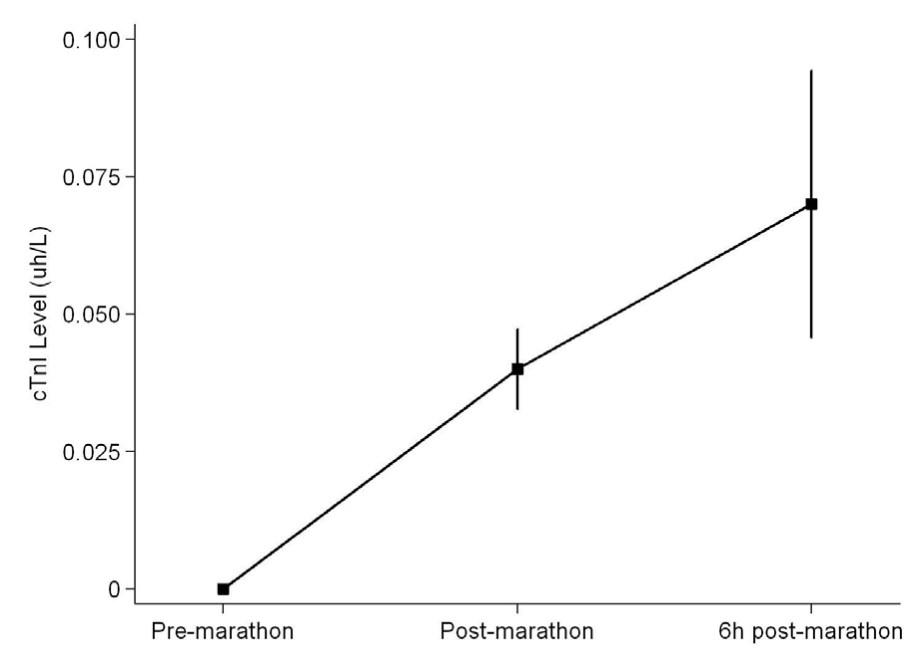
**Cardiac troponin I (cTnI) release pre-, immediately post- and 6 hrs post-completion of a marathon**. Values are mean (± SEM).

**Figure 2:**
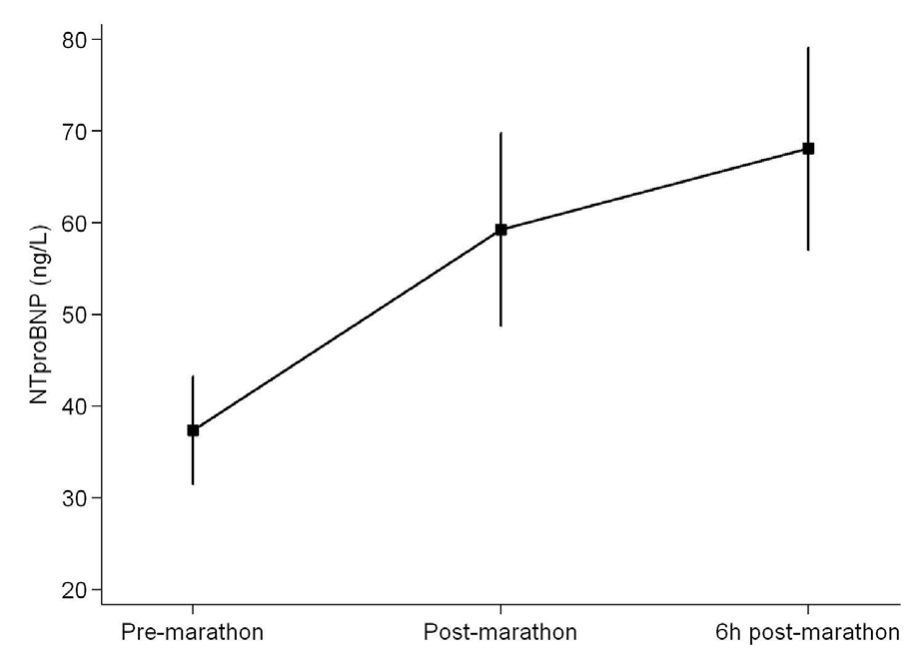
**NT-pro-B-type Natriuretic Peptide (NTproBNP) pre-, immediately post- and 6 hrs post-completion of a marathon**. Values are mean (± SEM).

No participant had elevations of TnI or NTproBNP at baseline. Eight participants were found to have cTnI elevations immediately post-marathon above the cut off level for acute myocardial infarction (AMI; ≥ 0.03 μg/L. cTnI was further elevated at 6 h post-marathon in 11 of the 17 runners in this study, p = 0.007, Tables 1 and 2, Figure 1. NTproBNP levels rose significantly from baseline at both time points post marathon, p = 0.002, Table 2, Figure 2.

**Table 2:** Data for indices of biological markers of cardiac damage pre-, post-, 6 hr post-marathon.

	Pre-marathon	Post-marathon	6 h Post-marathon	P value
cTnI (μg/L)	0.00	0.04 ± 0.008*	0.075 ± 0.024*	P = 0.002
NTproBNP (ng/L)	37.4 ± 5.9	59.3 ± 10.5*	68.1 ± 11*	P = 0.007

cTnI, cardiac troponin-I; NTproBNP, N terminal pro B type natriuretic peptide

*Significantly different from pre-marathon values (p < 0.05). Values are expressed as mean (± SD).

### CMR

**Figure 3:**
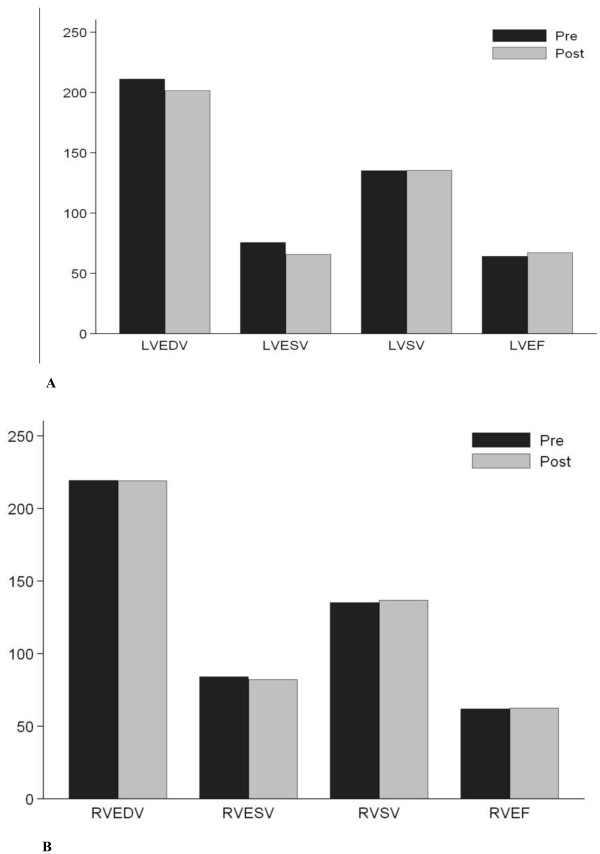
**CMR derived left (A) and right (B) ventricular volumes, stroke volume, and ejection fractions pre and 6 hours post marathon**. Values are mean (± SEM).

**Figure 4:**
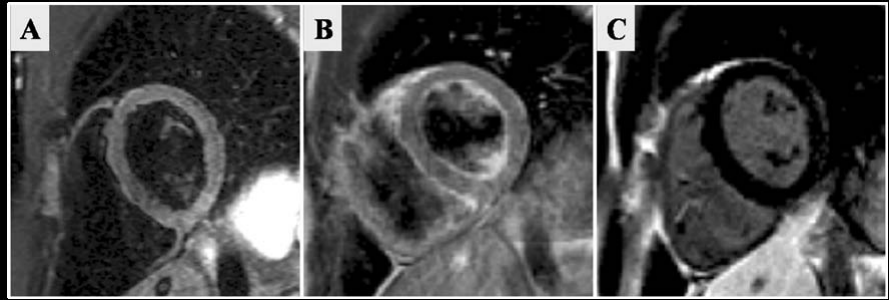
**Representative images of CMR acquisitions to detect myocardial oedema/inflammation (STIR) (A), hyperaemia (rGE) (B), and myocardial fibrosis (LGE) (C)**.

The LV end-diastolic and end-systolic volumes were reduced post marathon but the stroke volume was preserved (135.4 ± 5.0 vs. 135.5 ± 5.3 ml, P = NS) and a corresponding small increase in LV EF 6 hrs post-marathon reached significance (64.4% ± 1.0% vs 67.4% ± 1.2%; p = 0.014), Table 3, Figure 3a. No significant difference in the RV volumes, RV stroke volume or RV ejection fraction was seen post-marathon, Figure 3b. No patient demonstrated detectable focal or global myocardial oedema on pre and post marathon STIR imaging, Figure 4. Myocardial rGE pre and post contrast ratio was less than 45% in all, and none reached the rGE threshold of myocardium/skeletal muscle ratio of > 4.0, Table 4. Finally, no LGE was seen pre or post marathon in any participant. Four participants returned within 4 days to undergo a repeat CMR to look for any late changes in function or detectable tissue changes. None however demonstrated any LV or RV changes from baseline and none had detectable myocardial inflammation or fibrosis.

**Table 3:** Data indices for LV/RV function pre-, post- and 6 hr post-marathon.

CMR Volumetric Indices	Pre Marathon	6 Hrs Post-Marathon	P-Value
LVEDV (ml)	211 ± 8.4	201 ± 7.8	P = 0.002
LVESV (ml)	76 ± 4.3	66 ± 4.0	P = 0.002
LVSV (ml)	135 ± 5	135 ± 5.3	P = 0.955
LVEF (%)	64 ± 1	67 ± 1.2	P = 0.014
RVEDV (ml)	219 ± 9.9	219 ± 9.7	P = 0.933
RVESV (ml)	84 ± 5.4	82 ± 5.4	P = 0.571
RVSV (ml)	135 ± 5.5	137 ± 6.0	P = 0.399
RVEF (%)	62 ± 1.3	63 ± 1.4	P = 0.512

Values are expressed as mean ± SD.

**Table 4:** Myocardial oedema (STIR), hyperemia assessment (rGE), and fibrosis (LHE) pre and post marathon.

CMR Index	Pre Marathon	Post Marathon (SD)
Myocardial:Skeletal STIR Ratio	1.3 (SD 0.3)	1.4 (SD 0.3)
Percentage SI Increase in rGE Myocardium	30% (SD 8%)	31% (SD 9.6%)
rGE Myocardium:Skeletal Muscle Ratio	2.1 (0.9)	2.4 (1.0)
LGE	Nil	Nil

### Relationships between blood markers and myocardial damage

No significant correlations between biomarkers of cardiac damage (cTnI and NTproBNP) at any of the time points and measures of CMR systolic function, inflammation, hyperemia, or myocardial fibrosis were found.

## Discussion

This study demonstrates that exercise induced cardiac biomarker release is not associated with changes in biventricular systolic function or indeed any detectable myocardial damage (inflammation, oedema, hyperemia, or fibrosis) using current gold standard imaging modalities. The release of cardiac troponins (cTnT and cTnI) following prolonged exercise has been extensively documented and the high prevalence of cTnI and NTproBNP release following endurance exercise in this cohort is consistent with previous endurance studies [3-5]. The relationship between cardiac biomarkers and cardiac function following prolonged exercise continues to be a subject that generates much debate in the literature. Many studies have reported a lack of association between troponin release and functional changes, suggesting that they are distinctly separate phenomena [27]. Our data concur with these studies demonstrating no significant relationship between cTnI and NTproBNP and measures of cardiac function and support the hypothesis that alterations in cardiac function and the presence of cardiac biomarkers are unrelated.

The aetiology and clinical significance of post-exercise troponin release is yet to be elucidated. Shave et al. suggested that post-exercise release of troponin may represent either necrosis of cardiac myocytes leading to irreversible damage, or may be a transient and reversible change in membrane permeability of the myocyte [3]. The mechanisms for troponin release may come from the unbound pool found in the cardiomyocyte cytoplasm and may reflect a physiologic as opposed to a pathologic process [28]. It appears unlikely that the minor elevations in biomarkers of cardiac damage observed following prolonged endurance exercise indicate myocardial necrosis of sufficient magnitude to cause LV dysfunction. Furthermore, no single participant was found to meet criteria for myocardial inflammation or fibrosis following the marathon run. This suggests that elevated cardiac troponins represent reversible cardiomyocyte membrane damage that may reflect part of a remodelling process however further study is required to elucidate the mechanism.

A significant elevation in CMR EF was observed 6 hrs post-marathon, with no change in SV. This data contrasts with recent data that demonstrates persistent RV dysfunction post endurance exercise but is in agreement with the work of others [29-32]. In our work, the increase in EF noted after the marathon appears to be a compensatory increase to preserve stroke volume in the presence of reduced end-diastolic and end-systolic volumes. Whether this elevation in EF is a systolic rebound or over-performance issue remains to be confirmed. In the work by Mousavi et al, significant reductions in RVEF were noted in 14 participants studied following a marathon run. Important differences between this and our study should be highlighted which may account for the differences in our results and theirs. In particular we studied a male only population, whereas just over 40% of participants were female in the Mousavi study. It is also difficult to compare marathon completion times between both studies since the mean time for completion in their work of 245 minutes may reflect slower completion times for the female participants. We have provided a table to show completion times with peak biomarker release and CMR findings to avoid any confusion regarding individual differences in results.

## Limitations

Like many studies of this type, the numbers our cohort studied are small and the findings may not necessarily be applicable to larger populations. The spatial resolution of the CMR technique may also be insufficient to image small focal areas of myocardial inflammation or scarring caused by subclinical myocyte damage. We chose a 6 hour time point to perform the post marathon CMR and this may have influenced our findings. It is possible that changes in inflammation may not be detectable until after 6 hours but it was reassuring that 4 participants that returned within 4 days for a repeat CMR were not found to have any demonstrable structural or functional changes. The ratio used to evaluate myocardial oedema and hyperaemia was similar to that used in the Lake Louise criteria although we acknowledge that there were some differences in the protocol used with regard to the slice thickness. Work from our group suggests that this does not significantly alter the interpretation.

## Conclusions

This study showed that cardiac biomarkers suggestive of myocyte damage are elevated after a marathon, but these elevations are not associated with any detectable myocardial damage or acute changes in left or right ventricular function. Biomarkers of myocardial cell damage following an acute bout of prolonged exercise are not associated with functional changes using CMR and do not seem to be associated with any detectable myocardial inflammation, oedema, or scarring. This investigation provides further evidence for the careful consideration in assessing and treating symptomatic athletes following arduous exercise in the presence of elevated cardiac troponins and ECG anomalies.

## Abbreviations

CMR: Cardiovascular magnetic resonance; LV: left ventricle; BNP: Brain natriuretic peptide; CK: creatine kinase; CK-MB: creatine kinase-MB subunit; cTnI: cardiac troponin I; NT-proBNP: N-terminal brain natriuretic peptide; TSE: turbo spin secho; TR: repetition time; TE: echo time; RR: r-r interval; TI: inversion time; SA: short axis; FOV: field of view; LGE: late gadolinium enhancement; FLASH: fast low angle shot; ROI: region of interest; EF: ejection fraction; RV: right ventricle; STIR: short-tau inversion recovery; rGE: relative gadolinium enhancement

## Competing interests

Dr Pennell is a consultant to, and receives research support from Siemens. DR Pennell is a director of Cardiovascular Imaging Solutions. All other authors declared that they have no competing interests.

## Authors' contributions

GW, SKP and RO'H conceived the study, MW, GW, RO'H and SKP helped plan and design the study, MW, GW, RO'H, DJP, SP, DO, DG and RG collected the data, ROH, MW, GW, FA, and JW analysed the data, ROH and MW wrote the preliminary draft of the manuscript and all authors supplied comments and corrections, ROH and SKP are the guarantors. All authors read and approved the final manuscript.
